# A novel customizing knowledge graph evaluation method for incorporating user needs

**DOI:** 10.1038/s41598-024-60004-x

**Published:** 2024-04-26

**Authors:** Ying Zhang, Gang Xiao

**Affiliations:** Institute of Systems Engineering, Academy of Military Sciences (AMS), Beijing, 100107 China

**Keywords:** Knowledge graph quality assessment, Accuracy assessment, User requirement, Engineering, Mathematics and computing, Nanoscience and technology

## Abstract

Knowledge graphs are now widely used in various domains, including Question-and-answer systems, intelligent search and recommendation systems, and intelligent decision-making systems. However, knowledge graphs inevitably contain inaccurate and incomplete knowledge during the creation process, which leads to a reduction in the usefulness of knowledge graphs. Therefore, to assess the usefulness of knowledge graphs based on specific application requirements, quality assessment is particularly important. Among them, accuracy assessment, as a necessary dimension, reflects the degree of correctness of the triples. However, in the actual assessment process, the existing assessment methods do not consider the user’s needs and do not implement the concept of “Fitness for Use”. Meanwhile, it takes a lot of labor cost to evaluate the accuracy of large-scale knowledge graphs. Therefore, to ensure the accuracy of the assessment in a cost-saving way while meeting the needs of users, we propose and implement a novel accuracy assessment method that focuses on the requirements of users by designing an effective sampling method to obtain accurate assessment results that are more practical and instructive for users. Finally, the performance of our proposed method is evaluated by comparing it with the real accuracy rate, and the experimental results show that the accuracy rate obtained by the proposed method is very close to the real accuracy rate, and the sample size is minimized.

## Introduction

Knowledge graph quality assessment refers to the assessment of the triples in the knowledge graph in terms of accuracy, completeness, consistency, and redundancy^1–5^. More specifically, quality assessment can detect errors, inconsistencies, and outdated knowledge in the knowledge graph^6,7^, and at the same time, missing entities, relationships, or properties can be detected and measures can be taken to fill in these incomplete contents^1,8,9^. In addition, quality assessment can help identify and exclude untrustworthy information, increasing the user’s trust degree for the knowledge graph^10,11^. Further, quality assessment can also check the applicability of the current knowledge graph according to the needs of specific application scenarios^6,11,12^.

The main causes of quality problems in knowledge graphs are data problems, automated extraction problems, labeling problems, and quality assurance problems. Among them, data problems refer to data sources and incomplete data. Unreliable data sources, the existence of errors, and untimely updating will lead to the quality of the knowledge graph being affected^13–15^. In addition, building a complete knowledge graph requires a large amount of data covering the corresponding domains and topics. If domain data is missing, the completeness of the knowledge graph will be reduced^1,16^. The automated extraction problem refers to the unavoidable bias in processing complex statements or content with high ambiguity in the automated construction process, which consequently leads to the increase of misinformation in the knowledge graph^13,14,17,18^. The annotation problem refers to quality problems such as omissions, misjudgments, and inconsistencies caused by inaccurate understanding of concepts by annotators in some of the processes that require human involvement in annotation^19,20^. The quality assurance problem refers to the difficulty in maintaining a certain level of accuracy, completeness, and timeliness of the knowledge graph due to the inability to keep up with the subsequent upgrading and continuous updating of the knowledge graph^21^. To address the above potential quality problems, the following measures can usually be taken to improve quality. The first is to control the quality of data, ensuring that the data sources are reliable and accurate, and regularly updating and maintaining the data sources^14^. At the same time, diversify data sources and obtaining data from several different sources increase the coverage and accuracy of the knowledge graph^22,23^. Secondly, a combination of manual review and automated methods can also be used reasonably to reduce the introduction of erroneous information^24,25^. In addition, expert knowledge should be introduced to correct errors and inaccurate knowledge resulting from the construction process. Finally, the quality of the knowledge graph should be improved through subsequent continuous improvement and updating.

Currently, research on the quality assessment of knowledge graphs is still deepening, and it has shifted from the establishment of an assessment framework to the assessment and enhancement of a certain quality dimension. However, the concept of “Fitness for Use” has not yet been put into practice in the actual quality assessment process, and the definitions of some quality dimensions are directly derived from previous studies on data quality and linked data quality, which makes the conceptual system too general and macroscopic, and there are cases of crossover and overlap between the concepts. At the same time, the existing assessment method does not focus on the user’s point of view, which results in the assessment results not providing practical guidance to the user in selecting the applicable knowledge graph^21^. For example, when a knowledge graph meets the user’s required knowledge correctly, but the actual accuracy rate is low, it is difficult to judge whether this knowledge graph can be used by the user based on the accuracy rate alone. Therefore, we need to assess the quality of the knowledge graph in combination with the user’s actual needs, so that the results of the assessment have a certain reference value for the user. In this case, we need to design a reasonable evaluation method according to different usage requirements to obtain valuable evaluation results. In other words, users should evaluate the knowledge graph flexibly according to their own needs^26^, instead of simply calculating the average accuracy of the triples in the knowledge graph through a unified evaluation method. In addition, with the continuous development of technology, there are more and more large-scale knowledge graphs, so it is inevitable to spend a lot of time, computational cost, and labor costs to evaluate a dimension comprehensively and accurately. In particular, it is difficult for users to detect errors in the knowledge graph and to modify it accordingly before putting it into use in a short period and efficiently. Therefore, in such cases, it is important to obtain time-efficient evaluation results that are useful for users. With these considerations, we conducted the following study on the accuracy dimension of knowledge graphs.

In this study, we have explicitly proposed and implemented an innovative method for evaluating the accuracy of knowledge graphs. The core objective is to address the problem of traditional evaluation methods focusing too much on the intrinsic accuracy of the data while neglecting actual user needs. By systematically integrating user usage requirements into the evaluation framework, we emphasize enhancing the utility and effectiveness of the evaluation results for users, shifting the evaluation from mere data accuracy to close alignment with user practical application scenarios. Through the implementation of accuracy evaluation methods based on user needs, we have successfully tapped into user search behavior trends, ensuring that the evaluation results can closely align with actual user requirements. This initiative not only enables users to select more suitable knowledge graph resources according to their needs but also allows us to more accurately improve the accuracy level of knowledge graphs based on the evaluation results, to meet future diverse personalized demands. By analyzing in-depth the evolution trends of user knowledge demands, we provide a forward-looking perspective that guides the continuous improvement and development of knowledge graphs. In a series of experiments conducted on knowledge graphs, we employed an entity popularity-weighted clustering sampling method and found that by adjusting key parameters, specific threshold values significantly improved the accuracy and stability of the evaluation results, closely matching the actual accuracy rate, while significantly reducing the required sample size. These experimental results strongly confirm that our research objectives have been achieved; namely, through scientific method design and meticulous parameter adjustment, the evaluation process can meet actual user needs, significantly enhancing the applicability and accuracy of knowledge graph evaluation methods.

Our proposed evaluation method is based on the situation that user behavior remains relatively stable, particularly in specific application scenarios or domains, where user needs are generally fixed and predictable. During these circumstances, the search records we obtain are representative, and the evaluation results are indicative. Moreover, when the knowledge graph has achieved relative stability, analyzing user search data over a long period allows us to grasp long-term patterns and needs in user behavior, enabling better capture of changes and trends in user behavior. This, in turn, reflects sustained demand for specific entities in the knowledge graph, enhancing the representativeness of evaluation results while ensuring consistency and reliability. Additionally, in situations where human and time costs are limited, relying on past search records without overly pursuing accuracy for each entity can help us efficiently obtain evaluation results and save resources. The specific evaluation method involves crawling the access counts of high-traffic entities in the current knowledge graph. We allocate weights based on these access counts and then, on a per-entity cluster basis, iteratively extract samples until the error rate falls below a pre-set threshold determined by the user. At this point, the accuracy of the iteratively obtained samples is considered our sought-after accuracy. This method has been applied to the NELL, YAGO, and MOVIE knowledge graphs, yielding results highly close to the real accuracy rates. Our contributions are summarized as follows.

- We present a new methodology for assessing the accuracy of knowledge graphs, which, to our knowledge, is the first accuracy assessment study to incorporate user usage requirements.
- Results on a standard dataset show that our method uses a minimum number of samples while ensuring that the bias is as small as possible, yielding reliable accuracy assessment results of practical significance in an efficient and cost-saving way.

## Related work

Most of the existing research is centered around the assessment of the accuracy of knowledge graphs. Since the accuracy of knowledge graphs mainly depends on the knowledge sources and the process of constructing knowledge graphs, the assessment methods are mainly designed for the detection of error triples introduced by this process. As shown in Table 1, currently, common methods include the accuracy verification method using external data resources represented by Huaman et al.^27^, which mainly assesses the accuracy of the knowledge graph by setting corresponding weights for knowledge from different sources. Firstly, through entity matching, the same entities are sequentially obtained from different external data sources for comparison, and then the score of each entity is obtained. Similarly, the relevant information is obtained sequentially from different external data resources to score the triples associated with the entities, and the confidence scores of the triples are added to the scores of the corresponding entities to obtain the aggregated confidence scores of the entities. Second, some methods rely purely on model stacking to score triples. For example, Jia et al.^28^ evaluated the following three aspects in turn by stringing together three mini-models: the strength of correlation between entities, the satisfaction of translational invariance of relationship vectors, and the existence of reachable paths between head and tail entities. By relying on the model to make full use of the internal semantic information of the triples and the global inference information of the KG for a comprehensive assessment of the knowledge graph. Zhao et al.^31^ assessed the accuracy of the triples using the knowledge graph embedding method, by using the maximum expectation algorithm to combine the knowledge graph embedding model and the logic rules to encode the basic semantics of BioKG, a knowledge graph in the field of biomedicine, and to judge the correctness of its triple’s correctness. There are also evaluation methods that combine external knowledge, for example, Zhou et al.^30^ rely on crowdsourcing and neural networks to evaluate the quality of paths in the knowledge graph. The training dataset is first constructed by selecting more accurate, logical, and natural paths among the manually annotated paths, followed by using the resulting paths to train a neural network to predict potential path scores.

**Table 1:** Comprehensive comparison of knowledge graph accuracy assessment methods.

Method	Description	Characteristics	MS/EDR
Huaman et al.^27^	Calculate entity confidence based on weights	• Quantifying entity accuracy enables automatic evaluation of the knowledge graph • Requires manual setting of weights • Sensitive to the cost of large-scale knowledge sources • Needs to handle differences between different data sources	EDR
Jia et al.^28^	Concatenate small models to compute trustworthiness of entities and triples	• Capable of comprehensively considering accuracy from multiple aspects • High computational cost • High requirements for model performance, requiring fine-tuning	MS
Zhao et al.^31^	Integrating knowledge graph embedding models and logical rules for triple accuracy assessment	• Capable of considering global information of the knowledge graph • Able to leverage semantic information from embedding models • Designing and adjusting logical rules requires significant manual effort • Requires substantial computational resources support	MS
Ebeid et al.^32^	Utilizing Node2vec, Edge2vec, and similar methods to generate latent vector representations of entities, evaluating the correctness of triplets	• Can be flexibly applied to knowledge graphs in different domains • Requires ample training data • Sensitive to parameter tuning and model selection	–
Zhou et al.^30^	Combining crowdsourcing and neural networks to assess path quality	• Enhancing evaluation accuracy with external knowledge • Requires abundant human-annotated path data, relying on human assessment with subjectivity • Neural network models demand extensive training data	EDR

*MS stands for ‘Model stacking stand’ and EDR stands for ‘Incorporating external data resources’.

Although the above studies on knowledge graph accuracy measurement are continuously deepening, many studies focus on improving the accuracy results through time-consuming and labor-intensive methods such as model stacking and incorporating external data resources. The accuracy results obtained in the end are only slightly improved, and the degree of improvement is far from proportional to the cost incurred. In addition, the original purpose of the evaluation is to assist users in selecting a more appropriate, high-quality knowledge graph that better meets their needs. And the biggest quality problem is the lack of understanding of the requirements, and these methods ignore the actual needs of users in the actual evaluation process. As a result, the evaluation results are not different for any usage needs, and these methods treat every triple in the knowledge graph equally, even if it is outdated or will not be used in the future.

## Methodology

To solve the above problems, we propose a new sampling method EP-TWCS based on users’ usage requirements to evaluate the accuracy of knowledge graphs. Our evaluation method assigns different weights to different entities based on users’ usage needs, which makes the final accuracy evaluation results more representative. Meanwhile, in the process of sampling entity clusters, additional reference indicators are introduced to further ensure the precision of the measured accuracy and the use of as few samples as possible.

**Figure 1:**
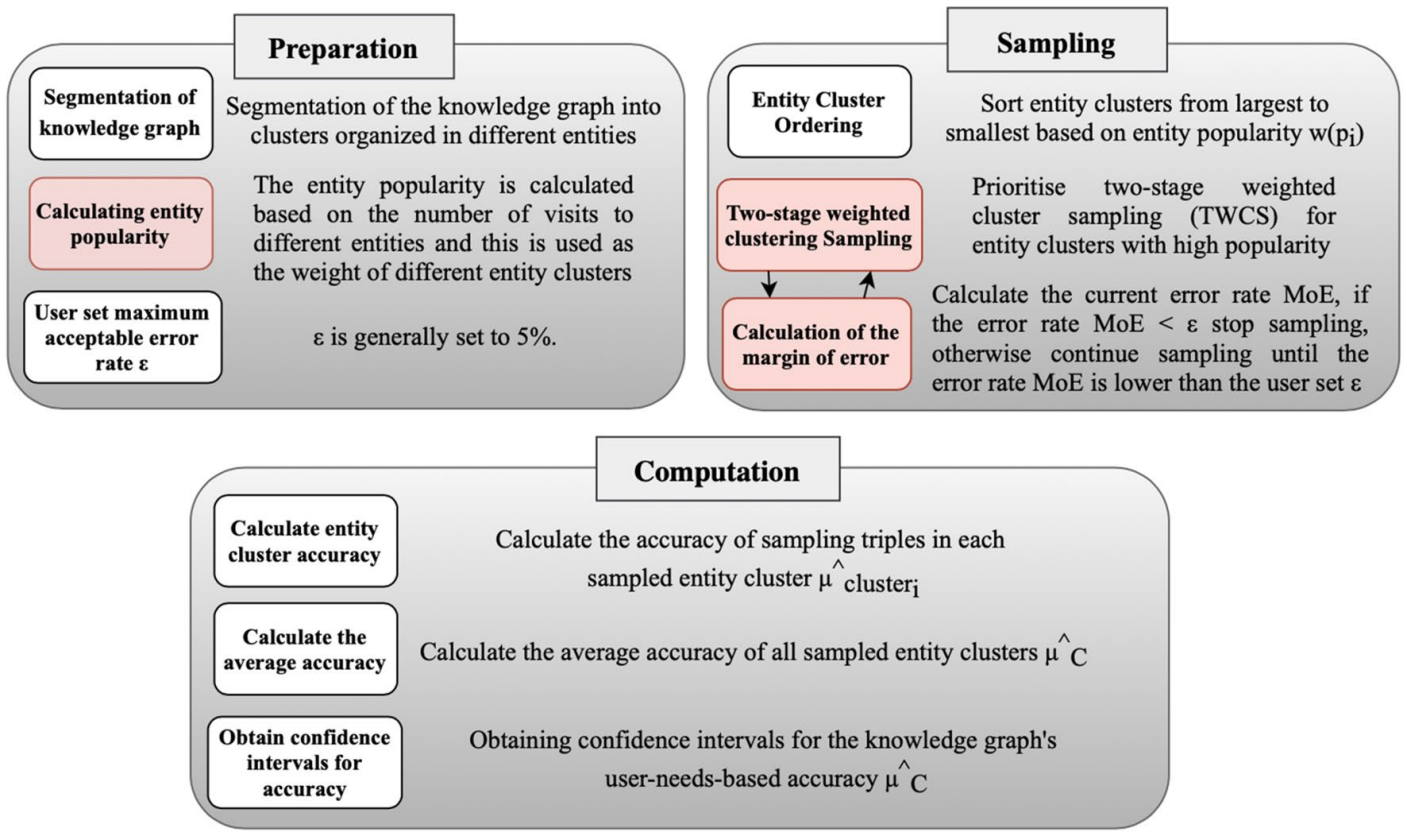
Flowchart of the assessment process, from left to right, the preparation phase, the sampling phase, and the calculation phase.

In this section, we describe the approach for knowledge graph accuracy assessment guided by user requirements, which is divided into three different steps, namely the preparation step, the sampling step, and the computation step, the details of the process are shown in Fig. 1. Initially, the knowledge graph is partitioned into clusters based on the concept of entity clusters, grouping them according to different entities. This approach assists us in effectively reducing the cost and time of triple evaluation by identifying and pinpointing entity identities. Particularly, when focusing on the same entity and its associated triples, evaluation efficiency can be significantly enhanced by minimizing redundant entity identification and utilizing centralized sources of information for batch fact verification. For instance, when triple evaluation is conducted without entity cluster classification, each triple requires entity matching for information retrieval and judgment. This repetitive search and query process, compared to evaluating the correctness of triples associated with a single entity at once, results in excessive time and labor costs. Next, we consider the user’s usage needs and take the accuracy rate of entities with high popularity that are frequently queried and accessed by the user as the user-need-based knowledge graph accuracy rate, to obtain the accuracy assessment results that are more meaningful to the user. The following are the specific steps for the accuracy assessment of the knowledge graph based on users’ needs.

### Preparation step

The preparation step requires segmenting the knowledge graph based on entity clusters, calculating entity popularity, and setting the maximum error rate ε acceptable to the user.

Segmentation of knowledge graph. Based on the above-mentioned concept of entity clusters the knowledge graph is segmented into clusters based on different entities.

Calculating entity popularity. The popularity of entities is calculated based on the degree of user demand for different entities. This is used as the weight parameter of different entity clusters in the knowledge graph, which is $w_{{p_{i} }}$, and the equations of $w_{{p_{i} }}$ is

1 $$ w_{{p_{i} }} = \frac{{s_{i} }}{S} $$

where $s_{i}$ is the number of times $entity_{i}$ has been queried in a specified period, and $S$ is the total number of times all the entities have been queried in a specified period. In addition to the popularity of the entity, it is also possible to consider some other relevant influences, such as the relevance of the entity to the user’s requirements, and the degree of time elapsed.

Set the error rate ε. ε is the maximum error rate acceptable to the user, which is usually set to 5% in general. In our study, we employ the concept of Margin of Error (MoE) to quantify the accuracy of estimates obtained during the iterative sampling process. MoE is a widely used standard in statistics to measure the potential deviation between sample statistics and corresponding population parameters. Essentially, it defines the reliable range of sample estimates, where a smaller MoE indicates a more precise estimation of population parameters and higher credibility of sample statistics. In our evaluation process, after each iteration of sampling, we calculate the size of MoE for that particular sample. Its mathematical expression is as follows:

2 $$ MoE = { }\frac{{{\text{z}} \times \sqrt {p \times \left( {1 - p} \right)} }}{\sqrt n } $$

where $n$ is the sample size, and $z$ corresponds to the z-value of the standard normal distribution at the selected confidence level, while $p$ represents the probability of correct triples occurring in the current sample. If the computed MoE is greater than the error rate ε, it indicates that the current sample size is insufficient to ensure the desired precision of the estimate, thus necessitating further expansion of the sampling size. Conversely, only when the MoE decreases to less than the error rate ε do we consider the current sample sufficient to reflect the characteristics of the population, thereby halting further sampling processes. Through such a control mechanism, we further ensure the robustness and reliability of the final computed results.

### Sampling step

To save labor and improve efficiency, we propose to prioritize the sampling method of entity clusters in the knowledge graph based on two-stage weighted cluster sampling (TWCS). The entity clusters are first sorted in descent order based on the entity popularity $$w_{{p_{i} }}$$ obtained in the previous step. Then, the clusters with high popularity are sampled first, and $${\text{min}}\left\{ {n_{{cluster_{i} }} ,n} \right\}$$ samples are randomly drawn from these clusters, where $$n_{{cluster_{i} }}$$ is the number of triples contained in the $$i$$ th entity cluster, and n is the maximum number of samples that can be drawn from each entity cluster by calculation. Then, the current error rate $$MoE$$ is calculated, if the error rate $$MoE$$ is lower than ε, then stop sampling, otherwise, continue sampling until the error rate $$MoE$$ is lower than the user-set ε. Eventually, the total sample capacity is $$ N_{TWCS}$$, and the deviation of the accuracy assessment result obtained by the third step does not exceed the pre-set ε.

### Computation step

When evaluating the accuracy of knowledge graphs through sampling methods, as only a portion of the samples can typically be observed, and samples represent only a fraction of the entire population, we need a method to estimate the difference between our estimated value and the true value. In this scenario, confidence intervals offer an effective approach to assess the uncertainty of accuracy estimation and provide a reliable means to quantify our confidence level in accuracy estimation^32^.

Firstly, the accuracy $\mu_{{cluster_{i} }}^{ \wedge }$ of each sampled entity cluster needs to be calculated, followed by computing the average accuracy $\hat{\mu }_{C}$ of all sampled entity clusters, $\hat{\mu }_{C}$ is computed as

3 $$ \hat{\mu }_{C} = { }\frac{1}{m}\mathop \sum \limits_{k = 1}^{m} \mu_{{cluster_{i} }}^{ \wedge } $$

where m represents the number of sampled entity clusters.

Next, under the given confidence level (or when the margin of error MoE is lower than ε), the confidence interval for the accuracy $\hat{\mu }_{C}$ of the knowledge graph based on user requirements is calculated. The width of the confidence interval is computed by multiplying the Z-score with the standard error, using the following formula

4 $$ \hat{\mu }_{C} \pm z_{\alpha /2} \sqrt {\frac{1}{{m\left( {m - 1} \right)}}\mathop \sum \limits_{k = 1}^{m} \left( {\mu_{{cluster_{i} }}^{ \wedge } - \hat{\mu }_{C} } \right)^{2} } $$

The resulting confidence interval provides a measure of uncertainty for estimating the accuracy of the knowledge graph. The calculated outcome represents the level of confidence we have in the estimated accuracy $\hat{\mu }_{C}$ of the knowledge graph based on user requirements, given a specific confidence level (or when the margin of error MoE is lower than ε). The confidence interval indicates the range within which the true accuracy is estimated to fall with a certain probability.

## Experiments

### Datasets

**Figure 2:**
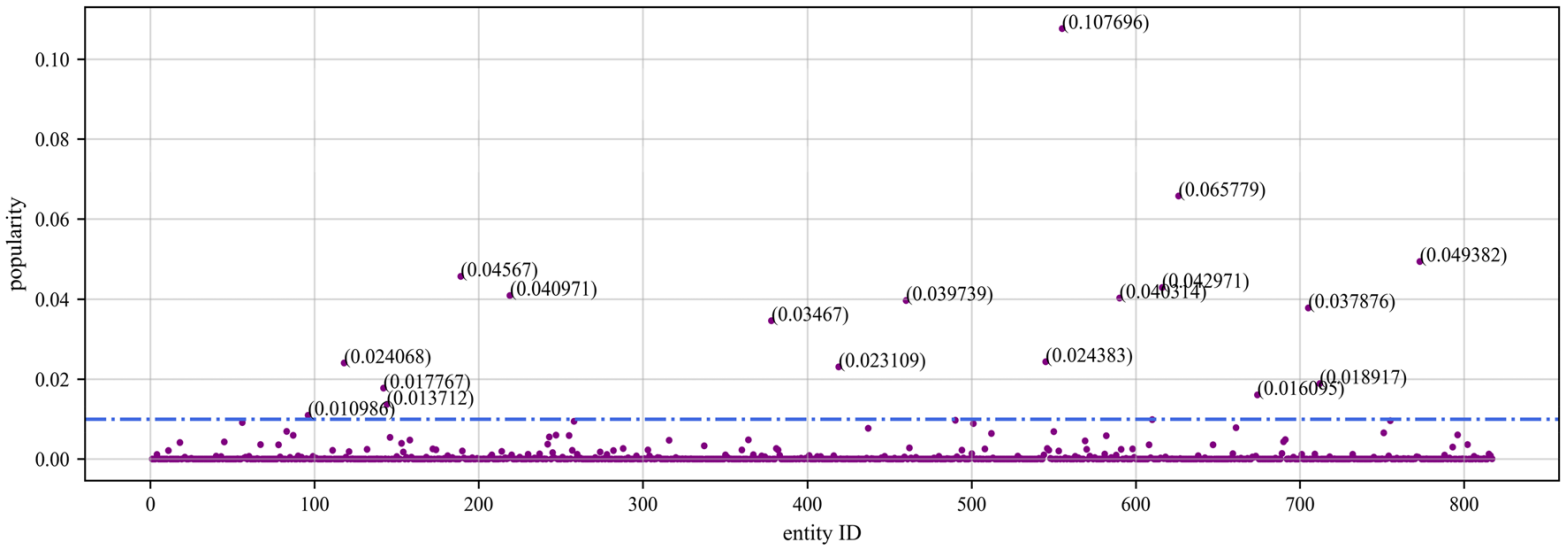
Scatter plot of NELL entity popularity.

**Figure 3:**
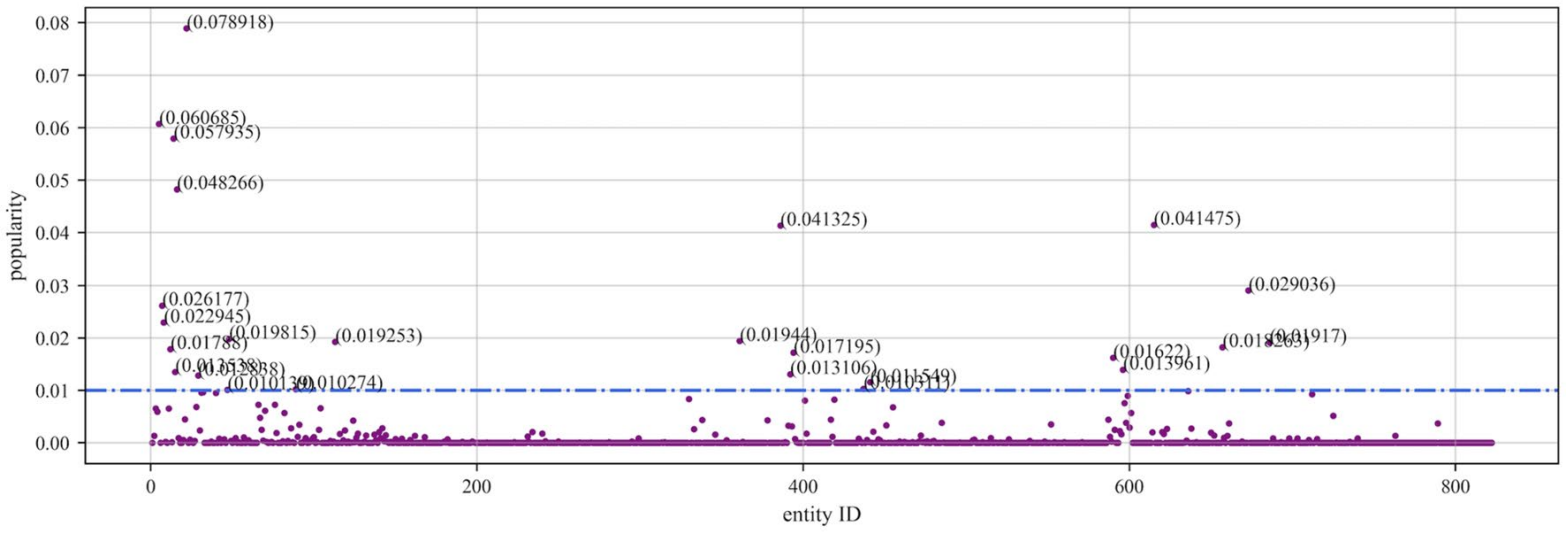
Scatter plot of YAGO entity popularity.

**Figure 4:**
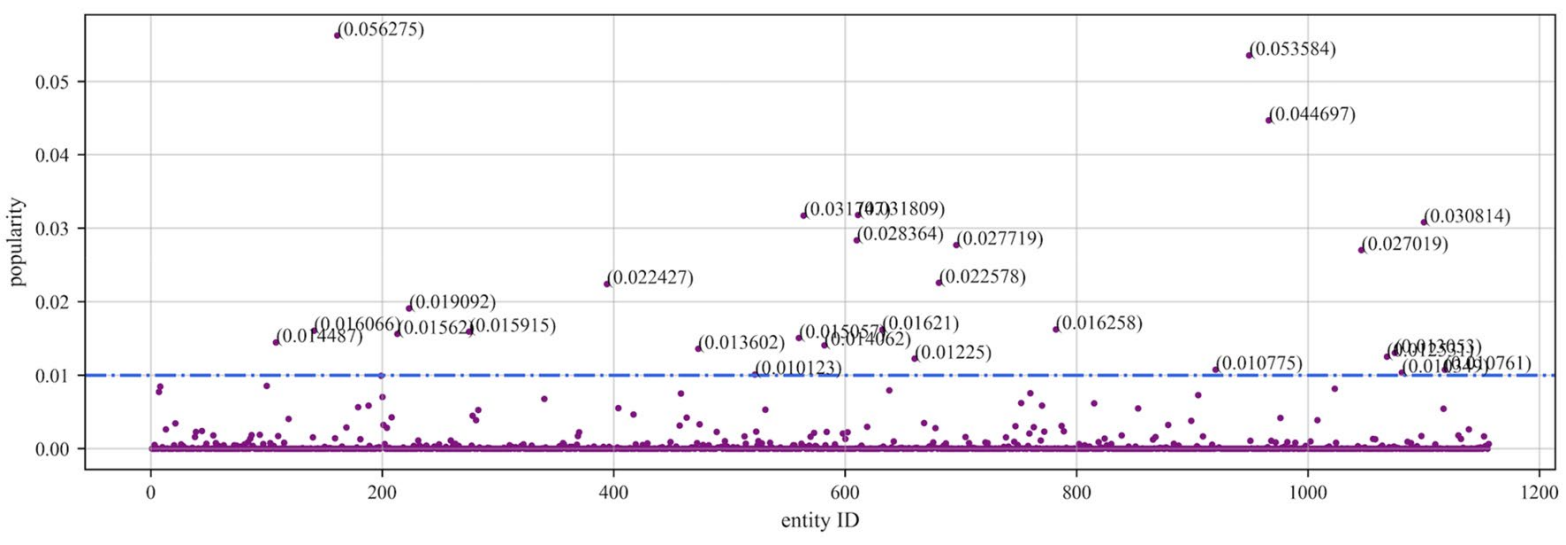
Scatter plot of MOVIE entity popularity.

To evaluate the proposed accuracy assessment method, we use the NELL knowledge graph. NELL is a domain knowledge graph extracted from NELL-Sports, which involves a total of 14 categories of sports, teams, athletes, coaches, etc., with 817 entities, where an entity corresponds to a maximum of 28 triples and a minimum of 1 triple. After manual annotation, the actual accuracy of the NELL knowledge graph is 91.3%. In addition, we also utilized the YAGO and MOVIE knowledge graphs. YAGO, a non-domain-specific subset of YAGO2, consists of 822 entities, with each entity having a maximum of 37 triples and a minimum of 1 triple, achieving an actual accuracy rate of 99%. The MOVIE knowledge graph comprises a total of 1156 movie entities, with each entity having a maximum of 3 triples and a minimum of 1 triple, with an actual accuracy rate of 90%. In addition, by modifying https://wikimedia.org/api/rest_v1/metrics/pageviews/perarticle/de.wikipedia/all-access/user/entity_name/daily/ start_time/end_time’s entity_name field as well as the start_time and end_time fields (in the format of YYYYMMDD), it is possible to crawl to obtain the frequency of different entities being accessed in Wikipedia during the corresponding period. We set the time in the range of 25 May 2023 to 1 June 2023. By following the above steps, we sequentially obtained the entity popularity distributions for the NELL, YAGO, and MOVIE knowledge graphs. As shown in Figs. 2, 3, and 4, we can observe that in knowledge graphs across different domains, there are quite a few entities that have had almost no query records during this period. The possibility of queries revolves mainly around certain important entities.

### Settings

All this experiment was conducted under the condition that ε is 5% and the confidence level is 95%. To avoid the situation where the triples with the high weights are all correct and cause the MoE to fall below ε prematurely, thus rendering the accuracy results obtained irrelevant, we sampled the top 1% of the prevalence entities beforehand to test the accuracy.

## Result and discussion

When different values of $n$ are taken, 100 experiments were conducted for the NELL, YAGO, and MOVIE knowledge graphs, yielding evaluation results as shown in Tables 2, 3, and 4 respectively (the data shown are in the case of stabilization of the accuracy). Furthermore, for reference, Tables 5, 6, and 7 respectively present the experimental results obtained using Simple Random Sampling (SRS), Random Cluster Sampling (RCS), Weighted Cluster Sampling (WCS), and Two-Stage Weighted Cluster Sampling (TWCS) methods on the above- mentioned knowledge graphs^29^.

**Table 2:** Resulting data from the EP-TWCS (95.5% true accuracy of sampled entity clusters).

Sampling method	Entity popularity Weighted clustering sampling (EP-TWCS) (Average cluster size = 2.28)						
Estimation	94.8%$\pm$ 2.9%	95.5%$\pm$ 3.0%	95.6%$\pm$ 2.9%	95.7%$\pm$ 2.9%	95.7%$\pm$ 2.8%	**95.5%**$\pm$** 3.0%**	95.5%$\pm$ 3.0%
	96.4%$\pm$ 3.5% (n = 3)	(n = 4)	(n = 5)	(n = 6)	(n = 7)	**(n = 8)**	(n = 9)
Sample Size	77 / 56	67	68	69	70	**67**	67

*Where $n$ is the maximum number of triple that can be sampled in an entity cluster.

**Table 3:** Resulting data from the EP-TWCS (96.72% true accuracy of sampled entity clusters).

Sampling method	Entity popularity weighted clustering sampling (EP-TWCS) (Average cluster size = 1.7)				
Estimation	0.974 $\pm 0.025$ (n = 2)	0.971 $\pm 0.017$ (n = 3)	0.969 $\pm 0.017$ (n = 4)	**0.968** $\pm 0.016$ **(n = 5)**	﻿ 0.968 $\pm 0.016$ (n = 6)
Sample size	39 / 26	32	31	**30**	﻿30

**Table 4:** Resulting data from the EP-TWCS (88.9% true accuracy of sampled entity clusters).

Sampling Method	Entity popularity Weighted clustering sampling (EP-TWCS) (Average cluster size = ﻿1.055)		
Estimation	0.889 $\pm$ 0.045	**0.889 ± 0.045 (n = 3)**	0.889 ± 0.045 (n = 4)
	0.896 $\pm$ 0.046 (n = 2)		
Sample size	153/144	**153**	153

**Table 5:** Results of previous experiments (NELL true accuracy of 91.3%).

Sampling method	Simple random sampling (SRS)	Random cluster sampling (RCS)	Weighted cluster sampling (WCS)	Two stage weighted cluster sampling (TWCS)
Estimation	91.5%$\pm$ 2.1%	90.5%$\pm$ 2.4%	91.6%$\pm$ 2.3%	91.6%$\pm$ 2.2%
Sample size	–	–	–	149 ± 47 [102,196]

**Table 6:** Results of previous experiments (YAGO true accuracy of 99%).

Sampling method	Simple random sampling (SRS)	Random cluster sampling (RCS)	Weighted cluster sampling (WCS)	Two stage weighted cluster sampling (TWCS)
Estimation	99.6% (96.7–100%)	98.9% (95.3%-100%)	99.2% (96.7%-100%)	99.2% (96.7–100%)
Sample Size	–	–	–	32 $\pm$ 5 [27,37]

**Table 7:** Results of previous experiments (MOVIE true accuracy of 90.0%).

Sampling method	Simple random sampling (SRS)	Random cluster sampling (RCS)	Weighted cluster sampling (WCS)	Two stage weighted cluster sampling (TWCS)
Estimation	90%	95%	93%	88%
Sample size	–	–	–	174

For NELL, during the experiment, we observed that when $$n$$ = 1, the sampling method is similar to simple random sampling, the average accuracy obtained in 100 experiments is 92.64%, and the result obtained for $$n$$ = 1 is the closest to the actual accuracy of 91.3% for NELL compared to the other values taken, and the number of samples required fluctuates between 39 and 176. The two accuracy confidence intervals obtained when $$ n $$ = 2 are (0.914, 0.971) and (0.928, 0.999), with sample means of 94.3% and 96.4%, respectively, and required sample sizes of 87 and 55, respectively. The two accuracy confidence intervals obtained when $$n$$ = 3 are (0.919, 0.977) and (0.93, 0.999), and the mean values of the accuracies obtained through 100 experiments are about 94.8% and 96.4%, and the number of samples required are 77 and 56, respectively. When $$n$$ = 4, 5, 6, 7, the accuracy confidence interval is fixed around (0.926, 0.985), the accuracy mean fluctuates around 95.6%, and the minimum number of samples required is 67. In this case, there is a slight fluctuation in the sample accuracy mean, which is mainly because the number of triples contained in some of the entity clusters does not satisfy greater than or equal to $$ n$$. Therefore, there is a certain range of normal fluctuation in the accuracy mean when the value of n becomes larger. Finally, when the value of $$n$$ is greater than or equal to 8, the accuracy of the knowledge graph obtained by Entity Popularity Weighted Clustering Sampling (EP-TWCS) gradually tends to be stable, and the average value of accuracy is 95.5%.

**Figure 5:**
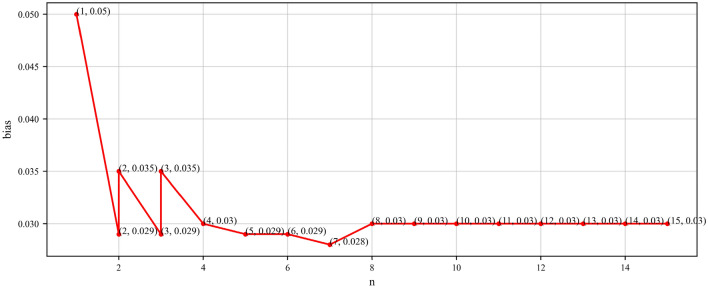
Line graph of $n$ taken as a function of bias (NELL).

To evaluate the accuracy of our method, we computed all triplets sampled from entity clusters in the NELL dataset, obtaining a true accuracy of 95.5%. Combining the experimental results presented in Table 2, it can be observed that the average accuracy measured in the evaluation gradually converges towards the true accuracy. The deviation from the actual accuracy decreases gradually with increasing n, reaching a maximum of 0.9%. Ultimately, when n is greater than or equal to 8, the experimentally obtained average accuracy aligns with the true value, with only 67 samples required. This indicates that our proposed method provides accuracy assessment results that are not only effective but also more valuable for users. Specifically, from the experimental results in Table 2, we also observed instability in accuracy assessment results, confidence intervals, and sample sizes when n is less than 8, showing some fluctuation and bias. However, these metrics stabilize when n is greater than or equal to 8. Hence, it is evident that n = 8 serves as the critical threshold for accuracy assessment in NELL. Based on this, we have discussed the different values of n, using the second half of the accuracy confidence interval (confidence interval = estimate ± critical value × error) as a reflection of the stability of n values. The results, as shown in Fig. 5, clearly illustrate that the relationship between n and bias roughly follows an “L”-shaped curve, with bias values gradually stabilizing as n increases, and stabilizing at a specific value when n = 8. Concurrently, referring to Table 5, accuracy stabilizes and approaches the true accuracy at n = 8.

Additionally, through researching relevant works, where^29^ has demonstrated effective practical application, we have conducted a comparative analysis to validate the effectiveness and fairness of our method. Compared to the methods presented in Table 5, our approach requires significantly fewer samples, much less than the 102 to 196 samples required in previous experiments. This comparison directly underscores the potential benefits of using our method for accuracy assessment in the NELL knowledge graph, particularly in terms of reducing the manual annotation effort and time consumption needed for annotating irrelevant triplets.

**Figure 6:**
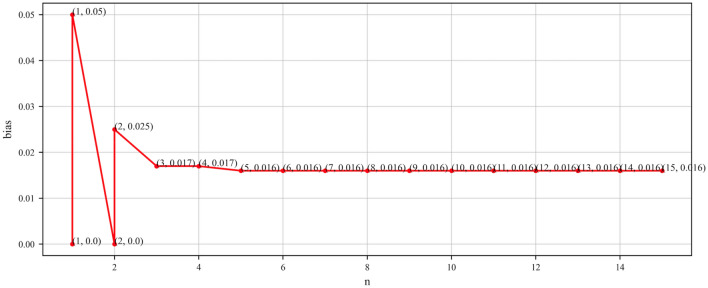
Line graph of $n$ taken as a function of bias (YAGO).

To further validate the generality and applicability of our proposed method, identical accuracy assessment experiments were conducted on two knowledge graphs, YAGO and MOVIE. Similarly, all triplets sampled from entity clusters in YAGO and MOVIE were computed, yielding true accuracies of 96.72 and 88.9%, respectively. Table 3 presents the accuracy assessment results obtained on YOGA, while Fig. 6 depicts the corresponding relationship between n and bias. From Table 3, it is evident that the deviation between the measured average accuracy and the actual accuracy gradually decreases with increasing n, eventually converging towards the true accuracy. Specifically, when n equals 5, the average accuracy, deviation, and required sample size stabilize. At this point, the accuracy of 96.8% is closest to the true accuracy of 96.72%, with only 30 samples required. Additionally, in conjunction with Fig. 6, it can be observed that the bias curve begins to stabilize when n is greater than or equal to 5. Therefore, for YAGO, n = 5 serves as the critical threshold for accuracy assessment, indicating that the accuracy measured at n = 5 is closest to the true accuracy. Ultimately, our proposed evaluation method for YAGO requires only 30 samples, compared to other evaluation methods presented in Table 6, which also avoids the sampling and manual annotation of irrelevant triples, achieving accurate assessment at minimal sample size and low cost (Table 7).

**Figure 7:**
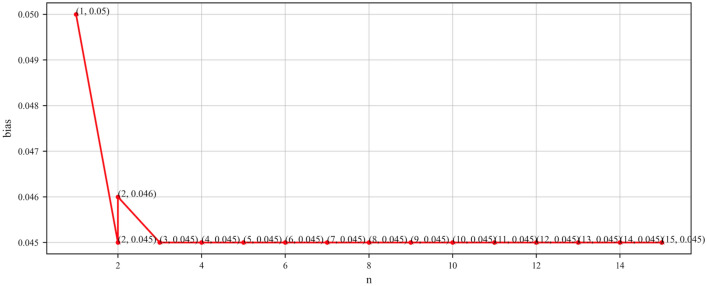
Line graph of $n$ taken as a function of bias (MOVIE).

For the domain-specific knowledge graph MOVIE, Table 4 presents the experimental results obtained on MOVIE, while Fig. 7 illustrates the corresponding relationship between n and bias. Based on Table 4, we can observe a pattern similar to what has been shown for NELL and YAGO. For the MOVIE knowledge graph, when n is greater than or equal to 3, the average accuracy, deviation, and required sample size begin to stabilize. At this point, the measured average accuracy infinitely approaches the true accuracy of 88.9%. Additionally, combining Fig. 7 reveals that when n is greater than or equal to 3, the bias curve starts to stabilize. Consequently, it can be concluded that n = 3 serves as the critical threshold for the accuracy assessment of the MOVIE knowledge graph, indicating that the accuracy measured at n = 3 is closest to the true accuracy. Similarly, compared to other methods in Table 7, we can also achieve reliable accuracy assessment using relatively fewer samples.

In general, across the NELL, YAGO, and MOVIE knowledge graphs, we observe a noticeable convergence in the trends of accuracy, sample size, and confidence interval as the value of n increases. At specific critical values of n (8, 5, and 3, respectively), the evaluation metrics reach a stable state closest to the actual accuracy. To further validate the effectiveness and universality of these critical n values, we delve into the evaluation results at different n values. Experimental data indicate that for each knowledge graph, the stability of the evaluation results significantly improves beyond their critical n values, minimizing the gap between accuracy and true accuracy. This phenomenon demonstrates the adaptability of our proposed evaluation method to specific characteristics of knowledge graphs and achieves evaluation goals under specific conditions.

Although the optimal n value for achieving the highest accuracy varies across different knowledge graphs, it does not diminish the practicality and universality of our method. The variation in n values is precisely determined by the differences in the inherent structural features and user requirements of each knowledge graph. This underscores the ability of the EP-TWCS method to adapt to various characteristics of knowledge graphs, minimizing sampling of irrelevant triples and unnecessary manual annotation costs while ensuring evaluation accuracy and effectiveness.

Furthermore, we note that in all experiments, as n increases to the critical point, bias exhibits a similar “L” shaped curve trend, indicating consistency in the performance of our evaluation method across different knowledge graphs, further enhancing the credibility of the results. Additionally, bias serves as a reliable indicator reflecting whether the currently obtained accuracy evaluation results are stable and accurate.

**Figure 8:**
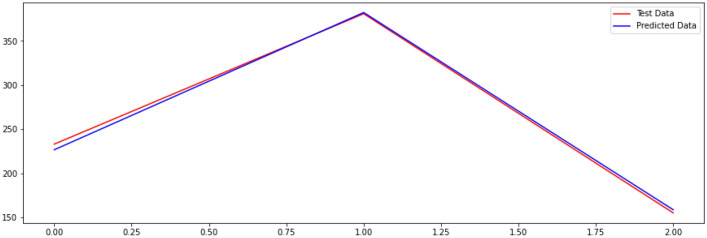
Plot fitting the relationship between ${y}_{1}$ and ${x}_{1},{x}_{2},{x}_{3},{x}_{4}$.

In addition, we consider the relationship between the average size of entity clusters and the sample size when the entity popularity is fixed. We evaluate the accuracy of knowledge graphs with different average sizes of entity clusters sequentially, where let $$x_{1}$$ be the average size of entity clusters, $$x_{2}$$ be the size of the largest clusters, $$x_{3}$$ be the size of the smallest clusters, $$x_{4}$$ be the number of triples contained in the current knowledge graph, $$y_{1}$$ be the number of samples drawn from the current knowledge graph. By collecting relevant data from experiments such as those mentioned above, we fit the relationships between $$x_{1} ,x_{2} ,x_{3} ,$$ and $$ x_{4}$$ with $$y_{1}$$. The root mean square error (RMSE) was used to assess the degree of model fit. By gradually narrowing down the RMSE values, the fitted graph as shown in Fig. 8 can be finally obtained. Ultimately, the relationship between the sample $$y_{1}$$ and $$x_{1} ,x_{2} ,x_{3} ,x_{4}$$ can be expressed by Eq. (5) (with each parameter rounded to two decimal places).

5 $$ y_{1} = 55.65 \times x_{1} - 4.31 \times x_{2} + 30.59 \times x_{3} - 0.07 \times x_{4} + 164.12 $$

In summary, when we know the average size of the entity clusters, the size of the largest cluster, the size of the smallest cluster, and the number of triples contained in the current knowledge graph, we can easily find the number of samples currently required for calculating the accuracy of the user-needs-based knowledge graph, by using the formulas mentioned above. Compared with the previous sampling method, our user-needs-centered computation method does not need to involve more entity clusters in sampling, which saves expensive manual annotation costs and ensures the accuracy of the evaluation results.

## Conclusion

In this paper, a new method for evaluating the accuracy of knowledge graphs tailored to user needs has been successfully developed. Through innovative expansions in sampling strategies, the method achieves a significant reduction in sample requirements and costs while maintaining high accuracy, thus effectively generating evaluation reports more practically valuable to users. This method is particularly suitable for application scenarios that prioritize user actual needs and seek efficient and cost-effective evaluation solutions. Specifically, the core contribution of this method lies in shifting the evaluation focus from purely the data level to the user’s actual needs, ensuring that the evaluation results closely align with user application scenarios. In the future, we plan to further integrate user requirements with other dimensions of knowledge graph quality to achieve comprehensive and efficient comprehensive evaluations of their quality. We are committed to exploring more economical and convenient approaches to optimize and enrich the knowledge content required by users while saving computing resources and manpower.

## Data Availability

The datasets used during the current study are available from the corresponding author on reasonable request.
